# Acute non-puerperal uterine inversion caused by a giant uterine leiomyoma with angioleiomyomatous features: A case report

**DOI:** 10.1016/j.crwh.2025.e00747

**Published:** 2025-09-02

**Authors:** Volkan Karatasli, Neslihan Bayramoglu, Ferhat Coskun

**Affiliations:** a Department of Gynecologic Oncology, Balıkesir Atatürk City Hospital, Balıkesir, Turkey; b Department of Gynecologic Oncology, Ankara Bilkent City Hospital, Ankara, Turkey; c Department of Pathology, Şanlıurfa Education and Research Hospital, Şanlıurfa, Turkey

**Keywords:** Angioleiomyomatous features, Case report, Leiomyoma, Uterine inversion

## Abstract

Non-puerperal uterine inversion is an extremely rare condition most often associated with uterine tumors. A 48-year-old woman presented with sudden vaginal bleeding and a large protruding mass. Emergency surgery revealed complete uterine inversion caused by a giant pedunculated mass arising from the uterine fundus. The tumor was excised vaginally, and the uterus was manually repositioned. Because multiple fibroids were also present and fertility was not desired, a total abdominal hysterectomy with bilateral salpingo-oophorectomy was performed. Histopathological examination revealed a leiomyoma with prominent angioleiomyomatous features, characterized by spindle cells surrounding thick-walled vascular structures and immunopositivity for desmin, smooth muscle actin, and progesterone receptor. The postoperative course was uneventful, and no recurrence was observed during 60 months of follow-up. This case highlights a rare morphological variant of uterine leiomyoma presenting with acute non-puerperal inversion and emphasizes the importance of early recognition and prompt surgical management.

## Highlights

• Non-puerperal uterine inversion is a rare gynecological emergency.
• A giant leiomyoma with angioleiomyomatous features caused acute inversion.
• Prompt surgical removal and hysterectomy ensured patient stabilization.
• Histopathology confirmed benign morphology with no recurrence on follow-up.

## 1 Introduction

Uterine inversion is most commonly observed as a postpartum complication, characterized by the inward collapse of the uterine fundus toward or through the cervix. In contrast, non-puerperal uterine inversion is rare and typically associated with intrauterine tumors, most frequently leiomyomas [1]. Rapid diagnosis and prompt surgical management are critical to preventing life-threatening hemorrhage and poor outcomes [2,3].

Angioleiomyomatous morphology represents a histological variant characterized by prominent vascular components within smooth muscle tissue. According to the WHO Classification of Female Genital Tumors (5th edition, 2020), such lesions are not recognized as a distinct uterine entity but are instead classified as leiomyomas with angioleiomyomatous features, despite their resemblance to angioleiomyoma of soft tissue [4]. Recognition of this morphology is important in diagnostic practice, particularly in rare clinical scenarios such as uterine inversion [5,6].

This report describes a case of acute non-puerperal uterine inversion caused by a giant pedunculated uterine leiomyoma with angioleiomyomatous features.

## 2 Case Presentation

A 48-year-old multiparous woman (gravida 5, para 5) with a body mass index of 21.1 kg/m^2^ presented to the emergency department with sudden-onset vaginal bleeding and a large mass protruding through the vaginal canal. She had no previous surgical history. She was hemodynamically unstable on admission. Physical examination revealed a lobulated, firm, highly vascular mass measuring approximately 20 cm, extending through the vagina. Pelvic ultrasonography failed to demonstrate the uterus in its expected anatomical location, raising suspicion of uterine inversion. Laboratory evaluation revealed a hemoglobin level of 6.8 g/dL. The patient was taken to emergency surgery for hemostatic management.

**Fig. 1:**
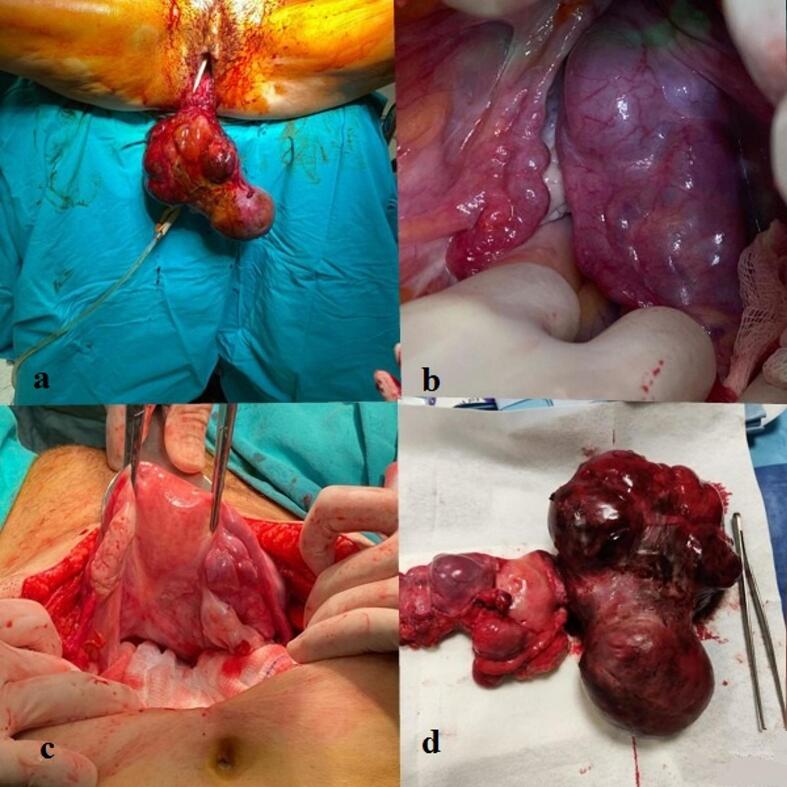
Intraoperative and macroscopic findings of acute non-puerperal uterine inversion caused by a giant uterine leiomyoma with angioleiomyomatous features. (a) Vaginal examination showing a large protruding mass; (b) Abdominal view demonstrating complete uterine inversion; (c) Restored uterine anatomy after manual repositioning; (d) Macroscopic specimen following hysterectomy.

Under general anesthesia, further examination confirmed complete uterine inversion extending into the vaginal canal (Fig. 1a). Laparotomy revealed downward displacement of the uterine fundus consistent with inversion (Fig. 1b). A pedunculated mass originating from the uterine fundus had prolapsed through the cervix and vagina. The pedicle was dissected and excised vaginally. After tumor removal, the uterus was manually repositioned into the abdominal cavity (Fig. 1c). Once the anatomical position was restored, multiple additional fibroids were observed on the uterine serosal surface and isthmus region. Given the patient's parity, lack of fertility desire, and presence of multiple fibroids, a total abdominal hysterectomy with bilateral salpingo-oophorectomy was performed (Fig. 1d). During surgery, the patient received seven units of erythrocyte suspension, five units of fresh frozen plasma, and two grams of fibrinogen.

**Fig. 2:**
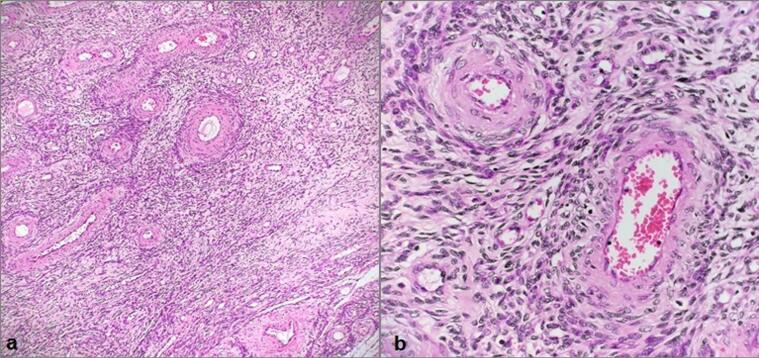
Histopathology of the excised tumor. (a) Tumor consisting of thick-walled vascular structures and spindle cells (H&Ex4); (b) Spindle cells forming cellularity around thick-walled vascular structures. Pleomorphism and mitotic activity were not observed (H&E x10).

On gross examination, the resected tumor measured 22 cm in its greatest dimension and appeared as a firm, vascular, encapsulated lesion. Additional fibroids were identified on the uterine serosa (5 cm) and near the isthmus (2.5 cm and 2 cm). Histopathological examination revealed interlacing bundles of spindle-shaped smooth muscle cells arranged around numerous thick-walled vascular channels (Fig. 2). No necrosis or significant mitotic activity was observed. The Ki-67 proliferation index was low (2–3 %).

**Fig. 3:**
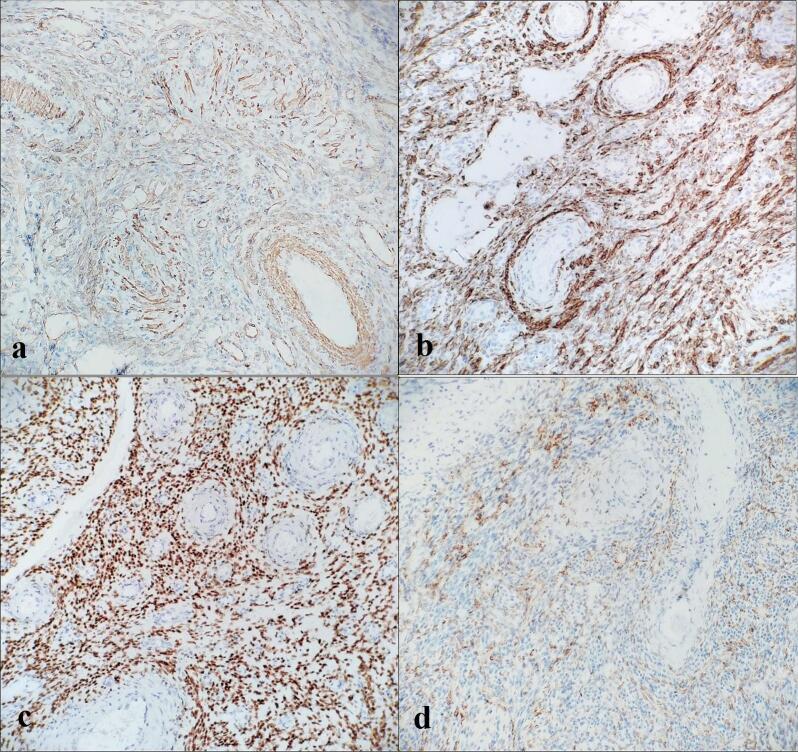
Immunohistochemistry of the tumor. (a) Smooth muscle actin positive, ×40; (b) Desmin positive, ×40; (c) Progesterone receptor positive, ×40; (d) CD10 negative, ×40.

Immunohistochemical staining demonstrated strong positivity for desmin, smooth muscle actin, and progesterone receptor, while negative for CD10 and HMB-45 (Fig. 3). These findings confirmed a diagnosis of leiomyoma with angioleiomyomatous features. No features of malignancy were observed.

The patient recovered uneventfully, was discharged with stable laboratory results, and remained asymptomatic throughout a 60-month follow-up.

## 3 Discussion

Non-puerperal uterine inversion is an uncommon gynecological emergency, with fewer than 200 reported cases [3]. Most cases are associated with submucosal leiomyomas, while malignancies are less frequent [2]. Rare vascular tumors such as hemangiomas have also been described as causative factors [7]. This case demonstrates that even a benign leiomyoma with angioleiomyomatous morphology can precipitate acute uterine inversion.

The mean age of patients reported with non-puerperal uterine inversion is approximately 46 years [3], which is consistent with the present case. Typical symptoms include acute pelvic pain, vaginal bleeding, and a protruding vaginal mass [2]. The condition should be suspected when the uterus is not palpable or visualized in its normal location. Magnetic resonance imaging (MRI) is considered highly valuable for diagnosis, as it allows visualization of the “U-shaped” uterine cavity and inverted fundus. Although MRI was not feasible in this patient due to hemodynamic instability, it may assist in timely diagnosis and differentiation from other pelvic masses in stable cases [2,3].

Immediate surgical management is essential. In this case, vaginal excision of the tumor and uterine repositioning were followed by hysterectomy, in line with common practice in multiparous women without fertility desire. Abdominal hysterectomy is the most frequently reported treatment [3].

Histologically, although the lesion resembled angioleiomyoma of soft tissue, the WHO Classification of Female Genital Tumors (5th edition, 2020) indicates that such lesions should be classified as leiomyomas with angioleiomyomatous features [4]. This interpretation is supported by the coexistence of other leiomyomas and progesterone receptor positivity. Recognition of this morphology remains of diagnostic importance, especially in rare presentations such as acute inversion. Predisposing factors for inversion include tumor size, fundal attachment, thinning of the uterine wall, cervical dilation, and multiparity, all of which were present in this case [2,3].

## 4 Conclusion

This case highlights an exceptionally rare cause of acute non-puerperal uterine inversion due to a giant leiomyoma with angioleiomyomatous features. Prompt diagnosis and surgical intervention are crucial. While hysterectomy is often the standard treatment in women without fertility desire, uterine-preserving approaches may be considered in younger patients. Long-term follow-up confirmed the benign nature of this lesion.

## Contributors

Volkan Karatasli contributed to patient care, conception of the case report, acquiring and interpreting the data, drafting the manuscript, undertaking the literature review and revising the article critically for important intellectual content.

Neslihan Bayramoglu contributed to patient care, interpreting the data and revising the article critically for important intellectual content.

Ferhat Coskun contributed to patient care, interpreting the data and revising the article critically for important intellectual content.

All authors approved the final submitted manuscript.

## Patient consent

Written informed consent was obtained from the patient for publication of the case report and accompanying images.

## Provenance and peer review

This article was not commissioned and was peer reviewed.

## Funding

No funding from an external source supported the publication of this case report.

## Declaration of competing interest

The authors declare they have no competing interest regarding the publication of this case report.
